# Influência do Tratamento com Doxorrubicina no Metabolismo da Heme em Cardiomioblastos: Estudo *In Vitro*

**DOI:** 10.36660/abc.20200662

**Published:** 2021-02-19

**Authors:** Mariana Gatto, Gustavo Augusto Ferreira Mota

**Affiliations:** 1 Departamento de Clínica MédicaFaculdade de Medicina de BotucatuUniversidade Estadual PaulistaBotucatuSPBrasilDepartamento de Clínica Médica, Faculdade de Medicina de Botucatu, Universidade Estadual Paulista - Unesp, Botucatu, SP - Brasil

**Keywords:** Doxorrubicina/uso terapêutico, Antraciclinas, Miócitos Cardíacos, Heme, Hemoglobina E/metabolismo

As últimas décadas vem sendo marcadas com potencial avanço no diagnóstico e terapia contra o câncer, gerando queda acentuada na mortalidade e aumento da sobrevida dos pacientes.^1^ A doxorrubicina é um fármaco que pertence à família das antraciclinas, uma classe de drogas anticâncer extraídas da estreptomicina.^2^ Embora o mecanismo de ação deste fármaco no câncer seja complexo, sabe-se que ele interfere na síntese de DNA e RNA além de induzir a produção de radicais livres que prejudicam a membrana celular e o DNA.^3^ Entretanto, muitos dos quimioterápicos utilizados no protocolo de tratamento das neoplasias induzem diversos efeitos colaterais, entre eles a toxicidade cardíaca e repercussões negativas no sistema vascular como isquemia trombolítica, hipertensão arterial, disfunção ventricular e insuficiência cardíaca.^1,4^ O efeito cardiotóxico da doxorrubicina é dose-dependente e o principal mecanismo de toxicidade é a indução de estresse oxidativo no miocárdio, com produção elevada de espécies reativas do oxigênio (ROS) e nitrogênio (RNS) que engatilham danos no DNA e desregulação de vários processos intracelulares.^5^

A molécula heme medeia a disponibilidade do ferro e é essencial para inúmeros processos biológicos nos organismos aeróbicos. No sistema cardiovascular desempenha papel crucial na defesa antioxidante, transdução de sinal, transporte de oxigênio, estocagem de hemoglobina e transporte de elétrons mitocondriais.^6^ No metabolismo da heme participam quatro enzimas mitocondriais e quatro citoplasmáticas. Para a síntese da heme, a glicina e a succinil coenzima A são condensadas no ácido δ-aminolevulínico (ALA) pela enzimas sintases de ácido aminolevulínico (ALAS), conhecidas como ALAS1 e ALAS2.^7^ Já a degradação da heme é realizada por enzimas denominadas heme oxigenase (HOX), que produzem monóxido de carbono, biliverdina e ferro. A isoenzima HOX-1 regula condições fisiológicas normais enquanto que a isoenzima HOX-2 protege células do estresse oxidativo.^8^ Por outro lado, estudos experimentais mostraram que elevação dos níveis da proteína heme estão relacionados com aumento do estresse oxidativo e toxicidade em cardiomiócitos.^9,10^ No entanto são escassos na literatura trabalhos que verificaram a influência das antraciclinas no metabolismo da proteína heme em cardiomiócitos.

Nesse sentido, o estudo publicado nos Arquivos Brasileiros de Cardiologia desta edição teve como finalidade explorar as mudanças na biossíntese da heme em linhagem de cardiomiócitos tratados com doxorrubicina.^11^ Para isso os autores avaliaram mudanças no perfil proteico e gênico das enzimas ALAS1, ALAS2, HOX-1 e HOX-2 em culturas de cardiomiócitos H9C2 tratadas com diferentes concentrações do fármaco por 24 horas.^11^ Os autores demonstraram que o tratamento com doxorrubicina aumentou significativamente os níveis de heme e que as enzimas ALAS1 e ALAS2 se comportaram de maneiras distintas. Menores doses de doxorrubicina inibiram a expressão de ALAS1 nos cardiomiócitos H9C2 e os autores sugerem que seja um mecanismo de feedback negativo, para prevenir a toxicidade celular induzida por níveis elevados de heme. Quando a dose do fármaco foi aumentada, a expressão de ALAS1 também aumentou, resultado corroborado por estudo prévio.^12^ Os níveis de ALAS2 foram decaindo conforme a dose da doxorrubicina era aumentada e os autores propuseram que este mecanismo é um efeito para contra regular o elevado nível de heme.

Em relação às enzimas HOX-1 e HOX-2, ambas apresentaram o mesmo comportamento a nível gênico, com expressão aumentada em relação ao controle quando doses maiores do fármaco foram utilizadas. A nível proteico foram detectados níveis aumentados de HOX-1 somente com as doses mais elevadas do medicamento enquanto HOX-2 apresentou aumento dos níveis de maneira dose-dependente.^11^ Os pesquisadores sugeriram que os resultados relacionados ao perfil proteico de HOX-1 e HOX-2 pode ser devido a diferenças nos mecanismos de regulação dessas enzimas. Além disso, existem poucos estudos que verificaram a relação de HOX-2 e doxorrubicina em cardiomiócitos.

Embora a doxorrubicina seja amplamente utilizada, muitos dos seus mecanismos cardiotóxicos permanecem incertos. A pesquisa em que se baseia esse minieditorial mostrou que o tratamento com esse medicamento em cardiomiócitos modula de maneira distinta os níveis gênicos e proteicos de enzimas cruciais da síntese e degradação das proteínas heme. Estudos futuros são necessários para elucidar o exato papel dessas enzimas em cardiomiócitos sob o tratamento com doxorrubicina.
