# Supplementary material for: Influência do Tratamento com Doxorrubicina no Metabolismo da Heme em Cardiomioblastos: Estudo In Vitro
Source: Arq Bras Cardiol. 2021 Feb 19;116(2):323–4. [Article in Portuguese] doi: 10.36660/abc.20200662 (PMC7909966; doi:10.36660/abc.20200662)
Supplement: Translation

# Translation: Influence of Doxorubicin Treatment on Heme Metabolism in Cardiomyoblasts: *An In Vitro* Study

**Authors:** Mariana Gatto, Gustavo Augusto Ferreira Mota

**Affiliations:** 1 BrasilDepartamento de Clínica Médica, Faculdade de Medicina de Botucatu, Universidade Estadual Paulista - Unesp, Botucatu, SP - Brasil

**Keywords:** Doxorubicin/therapeutic use, Anthracyclines, Myocytes, Cardiac, Heme, Hemoglobin E/metabolism

The last decades have been marked with potential advances in cancer diagnosis and therapy, leading to a decrease in mortality and increased patient survival.^1^ Doxorubicin is a drug that belongs to the anthracycline family, a class of anticancer drugs extracted from streptomycin.^2^ Although the mechanism of action of this drug in cancer is complex, it is known that it interferes with the synthesis of DNA and RNA in addition to inducing the production of free radicals that damage the cell membrane and DNA.^3^ However, many of the chemotherapeutic agents used in the neoplasms treatment protocols induce several side effects, including cardiac toxicity and negative repercussions in the vascular system such as thrombolytic ischemia, arterial hypertension, ventricular dysfunction and heart failure.^1,4^ The cardiotoxic effect of doxorubicin is dose-dependent, and the primary mechanism of toxicity is the induction of oxidative stress in the myocardium, with high production of reactive oxygen (ROS) and nitrogen (RNS) species that trigger DNA damage and deregulation of various processes intracellular.^5^

The heme molecule mediates the iron availability and is essential for numerous biological processes in aerobic organisms. In the cardiovascular system, it plays a crucial role in antioxidant defense, signal transduction, oxygen transport, hemoglobin storage, and mitochondrial electron transport.^6^ Four mitochondrial and four cytoplasmic enzymes participate in heme metabolism. For heme synthesis, glycine and succinyl coenzyme A are condensed into δ-aminolevulinic acid (ALA) by the enzymes aminolevulinic acid synthases (ALAS), known as ALAS1 and ALAS2.^7^ The degradation of heme is carried out by enzymes called heme oxygenase (HOX), which produces carbon monoxide, biliverdin, and iron. HOX-1 isoenzyme regulates normal physiological conditions while HOX-2 isoenzyme protects the cells from oxidative stress.^8^ On the other hand, experimental studies have shown that elevated heme protein levels are related to increased oxidative stress and toxicity in cardiomyocytes.^9,10^ However, few studies in the literature verified the influence of anthracyclines on heme protein metabolism in cardiomyocytes.

In this sense, the study published in the Arquivos Brasileiros de Cardiologia of this edition aimed to explore changes in heme biosynthesis in a cardiomyocytes cell lineage treated with doxorubicin.^11^ The authors evaluated changes in the protein and gene profile of the enzymes ALAS1, ALAS2, HOX-1, and HOX-2 in cultures of H9C2 cardiomyocytes treated with different concentrations of the doxorubicin for 24 hours.^11^ The authors demonstrated that treatment with doxorubicin significantly increased heme levels and that the enzymes ALAS1 and ALAS2 showed different behaviors. Lower doses of doxorubicin inhibited ALAS1 expression in H9C2 cardiomyocytes, and the authors suggest that it is a negative feedback mechanism to prevent cell toxicity induced by high levels of heme. When the dose of the drug was increased, ALAS1 expression also increased, a result corroborated by a previous study.^12^ ALAS2 levels decreased as the dose of doxorubicin was increased, and the authors proposed that this mechanism is an effect to counteract the high level of heme.

Regarding the HOX-1 and HOX-2 enzymes, both showed the same behavior at the gene level, with increased expression compared to the control when higher doses of the drug were used. At the protein level, increased levels of HOX-1 were detected only with the highest doses of the doxorubicin, while HOX-2 showed increased levels in a dose-dependent manner.^11^ The authors suggested that results related to the protein profile of HOX-1 and HOX-2 may be due to differences in the regulatory mechanisms of these enzymes. Also, few studies have verified the relationship between HOX-2 and doxorubicin in cardiomyocytes.

Although doxorubicin is widely used, many of its cardiotoxic mechanisms remain unclear. The research on which this short editorial is based has shown that treatment with doxorubicin in cardiomyocytes modulates gene and protein levels of crucial enzymes in the synthesis and degradation of heme proteins in a different way. Future studies are needed to elucidate the exact role of these enzymes in cardiomyocytes under treatment with doxorubicin.
